# Capabilities of hepatitis B surface antigen are divergent from hepatitis B virus DNA in delimiting natural history phases of chronic hepatitis B virus infection

**DOI:** 10.3389/fimmu.2022.944097

**Published:** 2022-07-25

**Authors:** Zhanqing Zhang, Wei Lu, Dan Huang, Xinlan Zhou, Rongrong Ding, Xiufen Li, Yanbing Wang, Weijia Lin, Dong Zeng, Yanling Feng

**Affiliations:** ^1^ Department of Hepatobiliary Medicine, Shanghai Public Health Clinical Center, Fudan University, Shanghai, China; ^2^ Department of Clinical Pathology, Shanghai Public Health Clinical Center, Fudan University, Shanghai, China

**Keywords:** hepatitis B surface antigen, chronic HBV infection, natural history, immune tolerance, locally estimated scatter plot smoothing regression, stratified analysis

## Abstract

**Objective:**

Quantitative hepatitis B surface antigen (HBsAg) and hepatitis B virus (HBV) DNA in the natural history of chronic HBV infection have not been rationally evaluated. This study aimed to re-characterize quantitative HBsAg and HBV DNA in the natural history phases.

**Methods:**

A total of 595 and 651 hepatitis B e antigen (HBeAg)-positive patients and 485 and 705 HBeAg-negative patients were assigned to the early and late cohorts, respectively. Based on the ‘S-shape’ receiver operating characteristic (ROC) curves, the HBeAg-positive sub-cohorts with possibly high HBV replication (PHVR) and possibly low HBV replication (PLVR) and the HBeAg-negative sub-cohorts with possibly high HBsAg expression (PHSE) and possibly low HBsAg expression (PLSE) were designated.

**Results:**

The areas under the ROC curve (AUCs) of HBsAg and HBV DNA in predicting HBeAg-positive significant hepatitis activity (SHA) in the early cohort, sub-cohort with PHVR, and sub-cohort with PLVR were 0.655 and 0.541, 0.720 and 0.606, and 0.553 and 0.725, respectively; those in the late cohort, sub-cohort with PHVR, and sub-cohort with PLVR were 0.646 and 0.501, 0.798 and 0.622, and 0.603 and 0.674, respectively. The AUCs of HBsAg and HBV DNA in predicting HBeAg-negative SHA in the early cohort, sub-cohort with PHSE, and sub-cohort with PLSE were 0.508 and 0.745, 0.573 and 0.780, and 0.577 and 0.729, respectively; those in the late cohort, sub-cohort with PHSE, and sub-cohort with PLSE were 0.503 and 0.761, 0.560 and 0.814, and 0.544 and 0.722, respectively. The sensitivity and specificity of HBsAg ≤4.602 log_10_ IU/ml in predicting HBeAg-positive SHA in the early cohort were 82.6% and 45.8%, respectively; those in the late cohort were 87.0% and 44.1%, respectively. The sensitivity and specificity of HBV DNA >3.301 log_10_ IU/ml in predicting HBeAg-negative SHA in the early cohort were 73.4% and 60.8%, respectively; those in the late cohort were 73.6% and 64.1%, respectively.

**Conclusion:**

Quantitative HBsAg and HBV DNA are valuable, but their capabilities are divergent in delimiting the natural history phases.

## Introduction

The accurate delimitation of the natural history phases and the assessment of the disease conditions of chronic hepatitis B virus (HBV) infection have important clinical implications for the valid management of patients and the rational use of antiviral drugs (1, 2).

Normatively, the natural history can be divided into four consecutive phases under the conventional criteria of serum hepatitis B e antigen (HBeAg) status, HBV DNA, and alanine transferase (ALT) levels, which are HBeAg-positive non-significant hepatitis activity (NSHA), HBeAg-positive significant hepatitis activity (SHA), HBeAg-negative NSHA, and HBeAg-negative SHA (3–6). However, according to the well-known evolution of HBV DNA levels in the natural history, the natural history can also be subdivided into six consecutive phases, which are established HBeAg-positive NSHA and SHA, re-established HBeAg-positive NSHA and SHA, and re-established HBeAg-negative NSHA and SHA. The established NSHA involves high levels of HBV DNA with normal ALT, and the established SHA relates to lowering high levels of HBV DNA with elevated ALT; the re-established NSHA involves low levels of HBV DNA with normal ALT, and the re-established SHA relates to elevating low levels of HBV DNA with elevated ALT. The subdivision of the natural history phases means that HBeAg-positive SHA involves two types of SHA that imply inversely evolving HBV replication levels. However, it should be noted that the re-established SHA is subordinate to the established SHA.

Commercialized quantitative HBV markers include hepatitis B surface antigen (HBsAg), HBeAg, hepatitis B core-related antigen (HBcrAg), antibodies against hepatitis B e antigen (anti-HBe), antibodies against hepatitis B core antigen (anti-HBc), HBV DNA, and HBV RNA (7). Among them, HBV DNA has been routinely used, and HBsAg has been widely promoted (3–6). However, although HBV DNA has been a key parameter for delimiting the natural history phases, its valid cutoffs, especially the one for delimiting HBeAg-positive NSHA and SHA, remain controversial (3–6); although HBsAg has been recommended as a parameter for delimiting the natural history phases, its quantitative criteria have not yet been established (3–6). Although HBsAg has an intrinsic association with HBV DNA, it does not necessarily correlate with HBV DNA, especially in HBeAg-negative NSHA and SHA (8–15). Sources of HBsAg involved HBV covalently closed circular DNA and HBV DNA integrated into the host genome (16–18). High levels of HBsAg and HBeAg may directly contuse the host’s immune response against HBV, which may relate to the maintenance of established HBeAg-positive NSHA (19–21).

The performance of HBsAg in delimiting HBeAg-positive NSHA and SHA has been evaluated. However, previous studies (8–15) have two limitations: first, they referred to the conventional criteria for delimiting the natural history phases covering HBV DNA, which means that they presumed the performance of HBV DNA is ‘optimal’; second, they only involved the un-stratified HBeAg-positive cohorts, which means that they ignored the potential interference of the re-established SHA. In addition, the capability of HBV DNA in delimiting HBeAg-negative NSHA and SHA has been recognized. However, previous studies (22–25) have also a limitation: they did not consider the latent impact of high levels of HBsAg on contusing the immune response. Those limitations suggest that previous studies may have misestimated the capabilities of HBsAg and HBV DNA in delimiting the natural history phases. Given the intrinsic association between HBsAg and HBV DNA, a rational evaluation of HBsAg in delimiting the natural history phases should only refer to serum biochemical and liver pathological criteria. Given the contusion of the immune response by high levels of HBsAg (19–21), a rational evaluation of HBsAg and HBV DNA in delimiting the natural history phases should involve the HBeAg-positive sub-cohorts with possibly high HBV replication (PHVR) and possibly low HBV replication (PLVR) and the HBeAg-negative sub-cohorts with possibly high HBsAg expression (PHSE) and possibly low HBsAg expression (PLSE).

The purpose of this study was based on early and late HBeAg-positive cohorts and their sub-cohorts with PHVR and PLVR, and early and late HBeAg-negative cohorts and their sub-cohorts with PHSE and PLSE, with reference only to the ‘biochemical and pathological criteria, to re-evaluate the performance of HBsAg and HBV DNA, which are the currently clinically accessible HBV markers, in delimiting the natural history phases.

## Materials and methods

### Study subjects

A total of 2,436 patients with full medical records, detailed liver pathology descriptions, and supporting laboratory data, who were hospitalized and underwent liver biopsy in Shanghai Public Health Clinical Center between January 2011 and August 2021, were involved in this retrospectively cross-sectional real-world study. Indications for liver biopsy involved in this study included the voluntary assessment of liver pathological conditions before the decision to accept or not to receive antiviral therapy under patients’ intention, and voluntary follow-up of liver pathological alterations after the decision not to receive antiviral therapy under patients’ preference. Written informed consent was obtained from all patients before liver biopsy. Patients with the following conditions were excluded (26): comorbid infection with other hepatotropic viruses (hepatitis A, C, D, and E virus), Epstein–Barr virus, cytomegalovirus; *Schistosomiasis japonica* disease, non-alcoholic fatty liver disease (steatosis >5%), excessive drinking (equivalent to ethanol, men >30 g/day, women >20 g/day), drug-induced liver injury, hyperthyroidism or hypothyroidism, gallstones or bile duct stones; antiviral therapy (nucleosides/nucleotides and interferon-α/peg-interferon-α), or hepato-protective therapy (glycyrrhizates, bicyclol/bifendate, matrine/oxymatrine, Chinese patent medicines, and Chinese medicine prescriptions) within 6 months prior to liver biopsy. Patients with fragmented biopsies or biopsy lengths less than 1.0 cm were also excluded.

Under the update of HBV DNA assays, which are described in section ‘Laboratory assays’, the patients involved were divided into four cohorts. A total of 595 and 485 patients hospitalized between January 2011 and August 2015 were involved in the early HBeAg-positive and HBeAg-negative cohort, respectively; 651 and 705 patients hospitalized between September 2015 and August 2021 were involved in the late HBeAg-positive and HBeAg-negative cohort, respectively.

This study was approved by the independent ethics committee of the Shanghai Public Health Clinical Center (ethical official number: 2013-K-008, 2018-S003-002) and conducted according to the guidelines of the 2013 Declaration of Helsinki.

### Laboratory assays

Fasting venous blood was collected in the morning within 1 week before and after liver biopsy and sent for laboratory tests within 30 to 60 min. Serum separation is performed by professionals in a clinical laboratory. HBsAg and HBeAg were measured by chemiluminescent microparticle immunoassay using an Abbott Architect i2000 automatic immunoassay system (Abbott Laboratories, Chicago, IL, USA) with corollary reagents (15, 26–28), the dynamic range of HBsAg is 0.05 to 250 IU/ml, and serum was diluted by 500-fold and re-measured if HBsAg exceeded the upper limit of detection; the lower limit of detection of HBeAg is 1.0 SCO. ALT in patients in the early cohorts was measured using a Hitachi 7600 automated biochemist analyzer (Hitachi, Tokyo, Japan) with corollary reagents; in patients in the late cohorts, ALT was measured using an Architect c16000 automatic biochemical analyzer (Abbott Laboratories, Chicago, IL, USA) with corollary reagents (15, 26–28).

HBV DNA in patients in the early cohorts was measured by dye-based qPCR assay using a Bio-Rad Icycler PCR System (Bio-Rad Laboratories, Inc., California, USA), the reagents were purchased from Qiagen Shenzhen Co. Ltd. (Shenzhen, China), and detection range is 500 to 5 × 10^7^ IU/ml (15, 27); in patients in the late cohorts, HBV DNA was measured by probe-based qPCR assay using a Roche LightCycler 480 qPCR system (Roche, Basel, Switzerland), the reagents were purchased from Sansure Biotech Inc. (Changsha, China), and detection range is 100 to 2 × 10^9^ IU/ml (26, 28).

### Pathological diagnoses

Ultrasound-guided percutaneous aspiration liver biopsy was performed, and the biopsy was placed directly into a pre-prepared plastic tube, snap-frozen, and transported for pathological diagnosis. The biopsies were processed within 36 h (26–28). Quality assessments and pathological diagnoses of the biopsies were conducted independently by a senior pathologist who was blinded to laboratory information. The liver pathological diagnosis referred to the Scheuer scoring system (29), in which pathological grade (Grade) is a description of the degree of necro-inflammation, with five grades from G0 to G4; pathological stage (Stage) is a description of the degree of fibrosis and alterations in architecture, with five stages from S0 to S4.

### Terminology designations

In this study, biochemically SHA was defined as ALT ≥40 IU/L (3, 4). Established and re-established pathologically SHA were defined as ‘Grade >G1 or Stage >S1’ and Grade >G1 (3–6), respectively.

Established HBeAg-positive NSHA was defined as ‘ALT <40 IU/L and Grade ≤G1 and Stage ≤S1’; established HBeAg-positive SHA was defined as ‘ALT ≥40 IU/L or Grade >G1 or Stage >S1’. Re-established HBeAg-positive and HBeAg-negative NSHA defined as ‘ALT <40 IU/L and Grade ≤G1’; re-established HBeAg-positive and HBeAg-negative SHA was defined as ‘ALT ≥40 IU/L or Grade >G1’.

HBeAg-positive SHA in the HBeAg-positive cohorts was specified as established or re-established HBeAg-positive SHA, which was the same definition as established HBeAg-positive SHA instead of re-established HBeAg-positive SHA; HBeAg-positive SHA in the sub-cohorts with PHVR was specified as established HBeAg-positive SHA; HBeAg-positive SHA in the sub-cohorts with PLVR was specified as re-established HBeAg-positive SHA. The SHA without limitation of HBeAg status in all the cohorts and sub-cohorts was specified as any type of SHA.

HBeAg-positive NSHA and SHA in unspecified cohorts or sub-cohorts have the same definition as established HBeAg-positive NSHA and SHA, respectively.

### Statistical analyses

MedCalc version 15.8 (MedCalc Software, Mariakerke, Belgium) was used for statistical analyses and graph creations. Locally estimated scatter plot smoothing (LOESS) regression was used to explore the evolution trends between ALT and HBsAg, HBeAg, and HBV DNA in the HBeAg-positive and HBeAg-negative cohorts (26). The receiver operating characteristic (ROC) curve was used to explore the latent ‘biphasic nature’ of HBV DNA and HBeAg in predicting SHA in the HBeAg-positive cohorts and of HBsAg in predicting SHA in the HBeAg-negative cohorts. The HBeAg-positive sub-cohorts with PHVR and PLVR and the HBeAg-negative sub-cohorts with PHSE and PLSE were designated, respectively, based on the ‘biphasic shape’ of the ROC curves of HBV DNA or HBV DNA and HBeAg in predicting SHA in the HBeAg-positive cohorts and of HBsAg in predicting SHA in the HBeAg-negative cohorts, respectively. ROC curve was used to evaluate the performance of HBsAg, HBeAg, and HBV DNA in predicting SHA in the HBeAg-positive cohorts and their sub-cohorts and of HBsAg and HBV DNA in predicting SHA in the HBeAg-negative cohorts and their sub-cohorts. Dependent-sample Hanley and McNeil non-parametric test was used to compare the differences in areas under the ROC curve (AUCs) between HBsAg, HBeAg, and HBV DNA in predicting SHA. p < 0.05 was considered statistically significant.

## Results

### Demographic, laboratory, and pathological characteristics of study subjects

The demographic, laboratory, and pathological data in the HBeAg-positive cohorts and their sub-cohorts, and the HBeAg-negative cohorts and their sub-cohorts, are summarized in **Table 1**. The bases and criteria for dividing the HBeAg-positive sub-cohorts and the HBeAg-negative sub-cohorts are described in section ‘Designation of hepatitis B e antigen-positive and hepatitis B e antigen-negative sub-cohorts’.

**Table 1:** Demographic, laboratory, and pathological characteristics of study subjects.

	HBeAg-positive cohort				HBeAg-negative cohort			
Variable*	Early (N = 595)	Late (N = 651)	*χ* ^2a^/Z^b^	p^#^	Early (N = 485)	Late (N = 705)	*χ* ^2a^/Z^b^	p^#^
Gender, male:female	391:204	395:256	3.386^a^	0.0658	315:170	411:294	5.338^a^	0.0209
Age, M (IQR)	32.0 (27.0 to 40.0)	34.0 (29.0 to 40.8)	3.294^b^	0.0010	43.0 (35.0 to 51.0)	43.0 (36.0 to 50.0)	0.109^b^	0.9134
ALT, M (IQR)	76.00 (36.00 to 182.00)	71.00 (38.00 to 160.00)	1.004^b^	0.3155	33.00 (21.00 to 73.25)	29.00 (19.00 to 59.25)	2.565^b^	0.0103
HBsAg, M (IQR)	3.911 (3.404 to 4.532)	3.865 (3.452 to 4.437)	0.641^b^	0.5215	3.110 (2.315 to 3.551)	3.270 (2.809 to 3.583)	3.216^b^	0.0013
HBeAg, M (IQR)	2.698 (1.608 to 3.092)	2.627 (1.472 to 3.101)	0.257^b^	0.7969				
HBV DNA^†^, M (IQR)	7.037 (5.680 to 7.699)	6.994 (5.488 to 7.748)	1.274^b^	0.2028	3.636 (2.699 to 4.995)	3.486 (2.031 to 4.761)	4.193^b^	<0.0001
Grade, 0:1:2:3:4	0:270:150:174:1	0:330:243:77:1	63.104^a^	<0.0001	332:76:77:0	524:123:58:0	16.740^a^	0.0002
Stage, 0:1:2:3:4	3:205:175:85:127	1:256:208:79:107	8.915^a^	0.0632	2:232:117:52:82	1:402:159:55:88	12.354^a^	0.0149
	Early HBeAg-positive sub-cohort				Late HBeAg-positive sub-cohort			
Variable *	PHVR (N = 423)	PLVR (N = 172)	*χ* ^2a^/Z^b^	p^#^	PHVR (N = 427)	PLVR (N = 224)	*χ* ^2a^/Z^b^	p^#^
Gender, male:female	272:151	119:53	1.292^a^	0.2557	257:170	138:86	0.124^a^	0.7248
Age, M (IQR)	31.0 (26.0 to 38.0)	35.0 (30.0 to 45.5)	4.824^b^	<0.0001	33.0 (29.0 to 38.0)	37.0 (32.0 to 44.0)	5.042^b^	<0.0001
ALT, M (IQR)	87.00 (45.00 to 203.50)	47.50 (28.00 to 126.00)	4.450^b^	<0.0001	91.00 (46.25 to 199.75)	44.00 (28.50 to 89.50)	7.759^b^	<0.0001
HBsAg, M (IQR)	4.234 (3.689 to 4.678)	3.385 (2.949 to 3.694)	12.273^b^	<0.0001	4.203 (3.801 to 4.625)	3.451 (3.124 to 3.710)	15.078^b^	<0.0001
HBeAg, M (IQR)	2.971 (2.392 to 3.123)	1.372 (0.607 to 2.267)	13.768^b^	<0.0001	3.035 (2.653 to 3.150)	1.107 (0.534 to 1.665)	19.677^b^	<0.0001
HBV DNA, M (IQR)	7.545 (6.959 to 7.699)	4.510 (3.681 to 5.353)	19.422^b^	<0.0001	7.531 (7.026 to 8.053)	5.081 (3.595 to 5.959)	18.875^b^	<0.0001
Grade, 0:1:2:3:4	0:191:115:117:0	0:79:35:57:1	5.999^a^	0.1117	0:224:156:47:0	0:106:87:30:1	3.588^a^	0.3096
Stage, 0:1:2:3:4	2:153:135:59:74	1:52:40:26:53	14.678^a^	0.0054	1:188:144:44:50	0:68:64:35:57	29.024^a^	<0.0001
	Early HBeAg-negative sub-cohort				Late HBeAg-negative sub-cohort			
Variable *	PHSE (N = 270)	PLSE (N = 215)	*χ* ^2a^/Z^b^	p^#^	PHSE (N = 305)	PLSE (N = 400)	*χ* ^2a^/Z^b^	p^#^
Gender, male:female	158:112	157:58	11.038^a^	0.0009	180:125	231:169	0.114^a^	0.7356
Age, M (IQR)	41.0 (33.0 to 50.0)	46.0 (36.0 to 53.0)	3.322^b^	0.0009	40.0 (33.0 to 47.0)	44.0 (38.0 to 52.0)	5.324^b^	<0.0001
ALT, M (IQR)	34.00 (21.00 to 75.00)	32.00 (21.00 to 70.00)	0.689^b^	0.4910	30.00 (19.00 to 61.50)	29.00 (20.00 to 58.50)	0.040^b^	0.9678
HBsAg, M (IQR)	3.505 (3.273 to 3.790)	2.212 (1.658 to 2.767)	18.930^b^	<0.0001	3.651 (3.492 to 3.836)	2.914 (2.140 to 3.177)	22.769^b^	<0.0001
HBV DNA, M (IQR)	4.200 (3.025 to 5.617)	2.821 (2.699 to 4.018)	7.316^b^	<0.0001	3.688 (2.507 to 5.155)	3.347 (2.000 to 4.444)	3.427^b^	0.0006
Grade, 0:1:2:3:4	0:188:40:42:0	0:144:36:35:0	0.447^a^	0.7998	0:225:55:25:0	0:299:68:33:0	0.129^a^	0.9377
Stage, 0:1:2:3:4	1:133:69:24:43	1:99:48:28:39	3.057^a^	0.5483	0:173:62:26:44	1:229:97:29:44	3.939^a^	0.4143

HBsAg, hepatitis B surface antigen; HBeAg, hepatitis B e antigen; HBV DNA, hepatitis B virus DNA; ALT, alanine transferase; Grade, pathological grade; Stage, pathological stage; PHVR, possibly high HBV replication; PLVR, possibly low HBV replication; PHSE, possibly high HBsAg expression; PLSE, possibly low HBsAg expression; M (IQR), median (interquartile range).

* Units of measurement: Age, years; ALT, IU/L; HBsAg, log_10_ IU/ml; HBeAg, log_10_ SCO; HBV DNA, log_10_ IU/m.

^#^ Early versus late, PHVR versus PLVR, and PHSE versus PLSE.

a Chi-squared test.

b Mann–Whitney U test.

^†^ Detection assays and reagents for the early cohorts and late cohorts are different, which are specifically described in section ‘Laboratory assays’.

### Correlation between hepatitis B surface antigen, hepatitis B e antigen, and hepatitis B virus DNA and alanine transferase, Grade, and Stage

**Figure 1:**
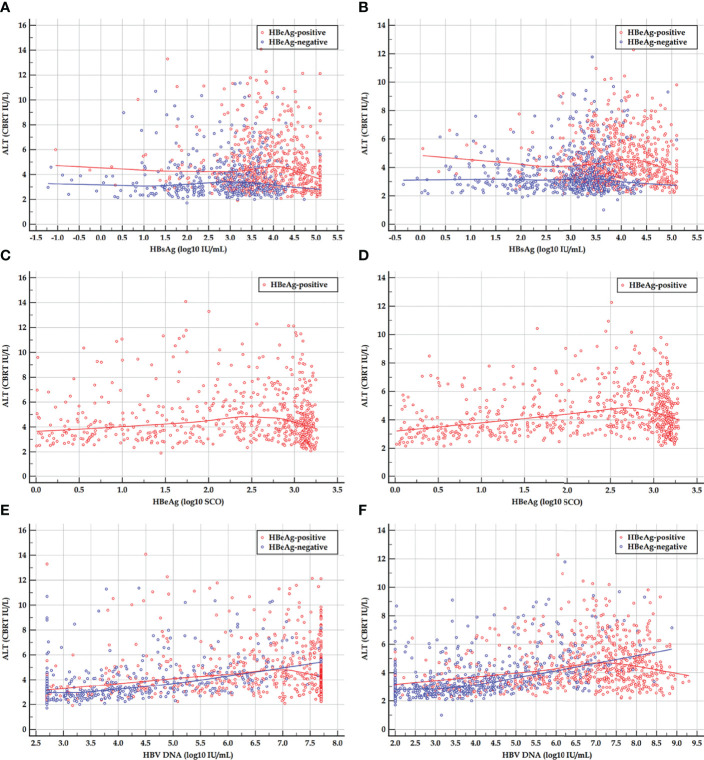
Scatter plots and LOESS regression curves between ALT and HBsAg, HBeAg, and HBV DNA in HBeAg-positive and HBeAg-negative cohorts. **(A, C, E)** Early HBeAg-positive and HBeAg-negative cohorts. **(B, D, F)** Late HBeAg-positive and HBeAg-negative cohorts. LOESS, locally estimated scatter plot smoothing; ALT, alanine transferase; HBsAg, hepatitis B surface antigen; HBeAg, hepatitis B e antigen; HBV DNA, hepatitis B virus DNA; CBRT, cubic root; log_10_, logarithm with base 10. The red and blue curves are LOESS regression trend lines in HBeAg-positive and HBeAg-negative cohorts. The degree of smoothing is controlled by a span of 60%.

The scatter plots and LOESS regression curves between ALT and HBsAg, HBeAg, and HBV DNA in the HBeAg-positive cohorts, and between ALT and HBsAg, and HBV DNA in the HBeAg-negative cohorts are displayed in **Figure 1**.

Spearman’s correlation coefficients between HBsAg, HBeAg, and HBV DNA and ALT, Grade, and Stage in the HBeAg-positive cohorts and their sub-cohorts, and between HBsAg, and HBV DNA and ALT, Grade, and Stage in the HBeAg-negative cohorts and their sub-cohorts are summarized in **Table 2**. The bases and criteria for dividing the HBeAg-positive sub-cohorts and the HBeAg-negative sub-cohorts are described in section ‘Designation of hepatitis B e antigen-positive and hepatitis B e antigen-negative sub-cohorts’.

**Table 2:** Spearman’s correlation coefficients between HBsAg, HBeAg, and HBV DNA and ALT, Grade, and Stage.

		HBeAg-positive cohort						HBeAg-negative cohort					
Correlated variable		Early (N = 595)		Late (N = 651)		Z	p^#^	Early (N = 485)		Late (N = 705)		Z	p^#^
x	y	rs	p	rs	p			rs	p	rs	p		
HBsAg	ALT †	−0.099	0.0159	−0.005	0.9063	−1.659	0.0971	0.026	0.5606	0.008	0.8420	0.304	0.7608
HBeAg	ALT †	−0.012	0.7660	0.150	0.0001	2.870	0.0041						
HBV DNA ^†^	ALT †	0.087	0.0340	0.201	<0.0001	2.050	0.0404	0.519	<0.0001	0.526	<0.0001	0.163	0.8707
HBsAg	Grade	−0.288	<0.0001	−0.265	<0.0001	0.438	0.6613	−0.057	0.2141	0.008	0.8286	1.100	0.2714
HBeAg	Grade	−0.272	<0.0001	−0.218	<0.0001	1.011	0.3121						
HBV DNA ^†^	Grade	−0.135	0.0010	−0.063	0.1058	1.280	0.2007	0.277	<0.0001	0.310	<0.0001	0.611	0.5415
HBsAg	Stage	−0.339	<0.0001	−0.333	<0.0001	0.119	0.9053	−0.071	0.1190	0.029	0.4432	1.693	0.0905
HBeAg	Stage	−0.367	<0.0001	−0.321	<0.0001	0.918	0.3586						
HBV DNA ^†^	Stage	−0.230	<0.0001	−0.188	<0.0001	0.773	0.4397	0.240	<0.0001	0.260	<0.0001	0.361	0.7184
		Early HBeAg-positive sub-cohort						Late HBeAg-positive sub-cohort					
Correlated variable		PHVR (N = 423)		PLVR (N = 172)		Z	p^#^	PHVR (N = 427)		PLVR (N = 224)		Z	p^#^
x	y	rs	p	rs	p			rs	p	rs	p		
HBsAg	ALT	−0.265	<0.0001	−0.158	0.0381	1.231	0.2183	−0.273	<0.0001	−0.214	0.0013	0.756	0.4495
HBeAg	ALT	−0.238	<0.0001	0.162	0.0341	4.458	<0.0001	−0.272	<0.0001	0.319	<0.0001	7.347	<0.0001
HBV DNA	ALT	−0.170	0.0005	0.333	<0.0001	5.685	<0.0001	−0.166	0.0006	0.359	<0.0001	6.548	<0.0001
HBsAg	Grade	−0.395	<0.0001	−0.153	0.0449	2.893	0.0038	−0.360	<0.0001	−0.161	0.0161	2.585	0.0097
HBeAg	Grade	−0.418	<0.0001	0.017	0.8251	5.075	<0.0001	−0.410	<0.0001	0.109	0.1024	6.570	<0.0001
HBV DNA	Grade	−0.269	<0.0001	0.161	0.0345	4.810	<0.0001	−0.145	0.0027	0.217	0.0011	4.418	<0.0001
HBsAg	Stage	−0.410	<0.0001	−0.101	0.1879	3.670	0.0002	−0.362	<0.0001	−0.099	0.1392	3.373	0.0007
HBeAg	Stage	−0.476	<0.0001	−0.030	0.6938	5.355	<0.0001	−0.424	<0.0001	0.144	0.0307	7.203	<0.0001
HBV DNA	Stage	−0.289	<0.0001	0.058	0.4519	3.903	0.0001	−0.166	0.0006	0.123	0.0671	3.510	0.0004
		Early HBeAg-negative sub-cohort						Late HBeAg-negative sub-cohort					
Correlated variable		PHSE (N = 270)		PLSE (N = 215)		Z	p^#^	PHSE (N = 305)		PLSE (N = 400)		Z	p^#^
x	y	rs	p	rs	p			rs	p	rs	p		
HBsAg	ALT	−0.065	0.2872	0.098	0.1542	1.776	0.0757	−0.046	0.4231	0.047	0.3477	1.219	0.2229
HBV DNA	ALT	0.617	<0.0001	0.426	<0.0001	2.882	0.0039	0.631	<0.0001	0.446	<0.0001	3.449	0.0006
HBsAg	Grade	−0.137	0.0244	0.040	0.5604	1.934	0.0531	−0.074	0.1982	0.043	0.3964	1.534	0.1249
HBV DNA	Grade	0.346	<0.0001	0.265	0.0001	0.972	0.3311	0.364	<0.0001	0.271	<0.0001	1.356	0.1751
HBsAg	Stage	−0.194	0.0014	0.142	0.0369	3.690	0.0002	−0.159	0.0054	0.113	0.0238	3.587	0.0003
HBV DNA	Stage	0.280	<0.0001	0.276	<0.0001	0.047	0.9624	0.265	<0.0001	0.257	<0.0001	0.112	0.9105

HBsAg, hepatitis B surface antigen; HBeAg, hepatitis B e antigen; HBV DNA, hepatitis B virus DNA; ALT, alanine transferase; Grade, pathological grade; Stage, pathological stage; PHVR, possibly high HBV replication; PLVR, possibly low HBV replication; PHSE, possibly high HBsAg expression; PLSE, possibly low HBsAg expression.

^#^ Early versus late, PHVR versus PLVR, and PHSE versus PLSE, Fisher Z test.

^†^ Detection methods and reagents for the early cohort and late cohort are different, which are specifically described in section ‘Laboratory assays’.

### Designation of hepatitis B e antigen-positive and hepatitis B e antigen-negative sub-cohorts

**Figure 2:**
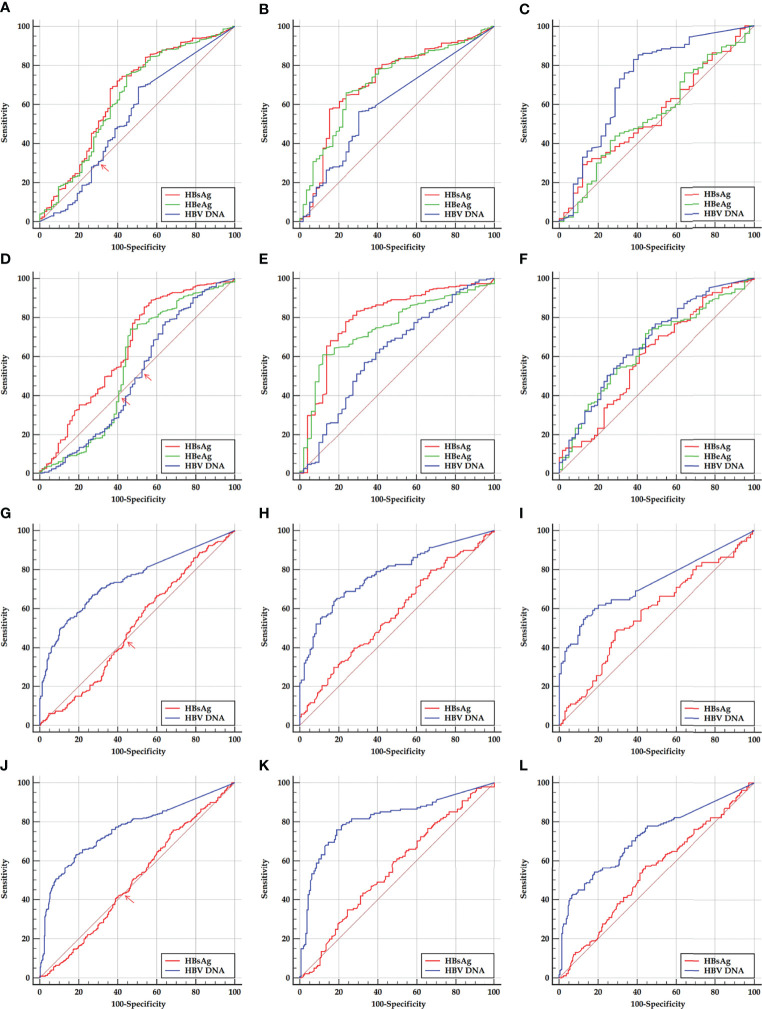
ROC curves of HBsAg, HBeAg, and HBV DNA in predicting SHA in HBeAg-positive and HBeAg-negative cohorts and their sub-cohorts. **(A, D)** Early and late HBeAg-positive cohorts. **(B, E)** Early and late HBeAg-positive sub-cohorts with PHVR. **(C, F)** Early and late HBeAg-positive sub-cohorts with PLVR. **(G, J)** Early and late HBeAg-negative cohort. **(H, K)** Early and late HBeAg-negative sub-cohorts with PHSE. **(I, L)** Early and late HBeAg-negative sub-cohorts with PLSE. ROC, receiver operating characteristic; HBsAg, hepatitis B surface antigen; HBeAg, hepatitis B e antigen; HBV DNA, hepatitis B virus DNA; PHVR, possibly high HBV replication; PLVR, possibly low HBV replication; PHSE, possibly high HBsAg expression; PLSE, possibly low HBsAg expression.

The ROC curves of HBsAg, HBeAg, and HBV DNA in predicting SHA in the HBeAg-positive cohorts are displayed in **Figures 2A, D**, and HBsAg and HBV DNA in predicting SHA in the HBeAg-negative cohorts are displayed in **Figures 2G, J**.

Among ROC curves of HBsAg, HBeAg, and HBV DNA in predicting SHA in the early HBeAg-positive cohort, the ROC curve of only HBV DNA displayed a ‘biphasic shape’; in the late HBeAg-positive cohort, the ROC curves of both HBeAg and HBV DNA displayed a ‘biphasic shape’. Among ROC curves of HBsAg and HBV DNA in predicting SHA in both the early and late HBeAg-negative cohorts, the ROC curves of HBsAg displayed a ‘biphasic shape’. A ‘biphasic shape’ ROC curve behaves as an ‘S-shape’ rather than a ‘C-shape’ curve (blue curve in **Figure 2A**, green and blue curves in **Figure 2D**, and red curves in **Figures 2G, J**).

Based on a criterion value for HBV DNA and HBeAg, and HBsAg, which depends on the sensitivity and specificity corresponding to an inflection point where the ‘S-shaped’ ROC curve intersects the diagonal reference line (red arrows in **Figures 2A, D, G, J**), a division point of HBV DNA and HBeAg, and HBsAg was determined, respectively, for dividing the possibly high and possibly low levels of HBV DNA and a division point of HBeAg in an HBeAg-positive cohort and the possibly high and possibly low levels of HBsAg in an HBeAg-negative cohort. The possibly high and possibly low levels of HBV DNA and HBeAg in the HBeAg-positive cohorts were used to designate PHVR and PLVR, respectively; the possibly high and possibly low levels of HBsAg in the HBeAg-negative cohorts were used to designate PHSE and PLSE, respectively.

In this study, HBV DNA >5.859 log_10_ IU/ml and HBV DNA ≤5.859 log_10_ IU/ml in the early HBeAg-positive cohort were designated as PHVR and PLVR, respectively; ‘HBV DNA >6.846 log_10_ IU/ml or HBeAg >2.333 log_10_ SCO’ and ‘HBV DNA ≤6.846 log_10_ IU/ml and HBeAg ≤2.333 log_10_ SCO’ in the late HBeAg-positive cohort were designated as PHVR and PLVR, respectively; HBsAg >3.033 log_10_ IU/ml and HBsAg ≤3.033 log_10_ IU/ml in the early HBeAg-negative cohort were designated as PHSE and PLSE, respectively; HBsAg >3.361 log_10_ IU/ml and HBsAg ≤3.361 log_10_ IU/ml in the late HBeAg-negative cohort were designated as PHSE and PLSE, respectively.

### Accuracy of hepatitis B surface antigen, hepatitis B e antigen, and hepatitis B virus DNA in predicting significant hepatitis activity

The ROC curves of HBsAg, HBeAg, and HBV DNA in predicting SHA in the HBeAg-positive cohorts and their sub-cohorts, and of HBsAg and HBV DNA in predicting SHA in the HBeAg-negative cohorts and their sub-cohorts are displayed in **Figure 2**; the corresponding AUCs are summarized in **Table 3**.

**Table 3:** AUCs of HBsAg, HBeAg, and HBV DNA in predicting SHA.

	Early HBeAg-positive cohort, prevalence = 86.05% (512/595)					Late HBeAg-positive cohort, prevalence = 87.10% (567/651)				
Variable	AUC	SE	95% CI	Z	p	AUC	SE	95% CI	Z	p
HBsAg	0.655^a,†^	0.0359	0.615 to 0.693	4.326	<0.0001	0.646^cd,†^	0.0365	0.608 to 0.683	4.004	0.0001
HBeAg	0.638^b,†^	0.0362	0.598 to 0.677	3.824	0.0001	0.554^c,†^	0.0412	0.515 to 0.593	1.312	0.1896
HBV DNA	0.541^ab,†^	0.0379	0.500 to 0.581	1.074	0.2829	0.501^d,‡^	0.0395	0.462 to 0.540	0.0181	0.9856
	Early HBeAg-positive sub-cohort with PHVR, prevalence = 86.05% (364/423)					Late HBeAg-positive sub-cohort with PHVR, prevalence = 88.06% (376/427)				
Variable	AUC	SE	95% CI	Z	p	AUC	SE	95% CI	Z	p
HBsAg	0.720^e,†^	0.0374	0.674 to 0.762	5.876	<0.0001	0.798^g,†^	0.0358	0.756 to 0.835	8.327	<0.0001
HBeAg	0.717^f,†^	0.0352	0.672 to 0.760	6.180	<0.0001	0.750^h,†^	0.0334	0.706 to 0.790	7.484	<0.0001
HBV DNA	0.606^ef,†^	0.0395	0.557 to 0.653	2.677	0.0074	0.622^gh,†^	0.0443	0.574 to 0.668	2.758	0.0058
	Early HBeAg-positive sub-cohort with PLVR, prevalence = 75.58% (130/172)					Late HBeAg-positive sub-cohort with PLVR, prevalence = 72.77% (163/224)				
Variable	AUC	SE	95% CI	Z	p	AUC	SE	95% CI	Z	p
HBsAg	0.553^i,†^	0.0498	0.475 to 0.629	1.062	0.2883	0.603^†^	0.0439	0.536 to 0.668	2.353	0.0186
HBeAg	0.547^j,‡^	0.0515	0.470 to 0.623	0.917	0.3592	0.644^‡^	0.0407	0.578 to 0.707	3.543	0.0004
HBV DNA	0.725^ij,‡^	0.0503	0.652 to 0.790	4.477	<0.0001	0.674^‡^	0.0409	0.609 to 0.735	4.257	<0.0001
	Early HBeAg-negative cohort, prevalence = 51.13% (248/485)					Late HBeAg-negative cohort, prevalence = 46.24% (326/705)				
Variable	AUC	SE	95% CI	Z	p	AUC	SE	95% CI	Z	p
HBsAg	0.508^k,†^	0.0265	0.462 to 0.553	0.297	0.7663	0.503^l,‡^	0.0218	0.465 to 0.540	0.129	0.8970
HBV DNA	0.745^k,‡^	0.0223	0.704 to 0.783	10.957	<0.0001	0.761^l,‡^	0.0186	0.728 to 0.792	14.058	<0.0001
	Early HBeAg-negative sub-cohort with PHSE, prevalence = 51.11% (138/270)					Late HBeAg-negative sub-cohort with PHSE, prevalence = 46.23% (141/305)				
Variable	AUC	SE	95% CI	Z	p	AUC	SE	95% CI	Z	p
HBsAg	0.573^m,†^	0.0347	0.512 to 0.633	2.108	0.0350	0.560^n,†^	0.0329	0.502 to 0.616	1.815	0.0696
HBV DNA	0.780^m,‡^	0.0280	0.726 to 0.828	10.013	<0.0001	0.814^n,‡^	0.0259	0.766 to 0.856	12.122	<0.0001
	Early HBeAg-negative sub-cohort with PLSE, prevalence = 51.16% (110/215)					Late HBeAg-negative sub-cohort with PLSE, prevalence = 46.25% (185/400)				
Variable	AUC	SE	95% CI	Z	p	AUC	SE	95% CI	Z	p
HBsAg	0.577^o,‡^	0.0392	0.508 to 0.644	1.960	0.0500	0.544^p,‡^	0.0289	0.494 to 0.593	1.513	0.1303
HBV DNA	0.729^o,‡^	0.0346	0.665 to 0.788	6.630	<0.0001	0.722^p,‡^	0.0259	0.675 to 0.765	8.559	<0.0001

AUC, area under receiver operating characteristic curve; SHA, significant hepatitis activity; SE, standard error; 95% CI, 95% confidence interval; HBsAg, hepatitis B surface antigen; HBeAg, hepatitis B e antigen; HBV DNA, hepatitis B virus DNA; PHVR, possibly high HBV replication; PLVR, possibly low HBV replication; PHSE, possibly high HBsAg expression; PLSE, possibly low HBsAg expression.

^a–p^ Dependent-sample Hanley and McNeil non-parametric test: a, Z = 3.776, p = 0.0002; b, Z = 3.148, p = 0.0016; c, Z = 2.820, p = 0.0048; d, Z = 5.221, p < 0.0001; e, Z = 3.106, p = 0.0019; f, Z = 2.589, p = 0.0096; g, Z = 4.389, p < 0.0001; h, Z = 2.693, p = 0.0071; i, Z = 2.542, p = 0.0110; j, Z = 2.998, p = 0.0027; k, Z = 8.421, p < 0.0001; l, Z = 10.088, p < 0.0001; m, Z = 4.846, p < 0.0001; n, Z = 6.228, p < 0.0001; o, Z = 3.202, p = 0.0014; p, Z = 5.312, p < 0.0001.

^†^ Smaller observed criterion values indicate lower sensitivity and higher specificity for predicting SHA.

^‡^ Larger observed criterion values indicate lower sensitivity and higher specificity for predicting SHA.

An optimal cutoff and a tradeoff cutoff were determined, respectively, based on the maximum sum (or Youden’s index) and minimum difference between specificity and sensitivity. With reference to one with the highest sensitivity in the optimal cutoff and tradeoff cutoff, a practical cutoff was chosen, which is easy to remember and the sensitivity of which is close to and higher than the highest sensitivity of the optimal cutoff and tradeoff cutoff. The optimal cutoffs, tradeoff cutoffs, practical cutoffs, and corresponding diagnostic parameters are summarized in **Table 4**.

**Table 4:** Cutoffs and corresponding diagnostic parameters of HBsAg, HBeAg, and HBV DNA in predicting SHA.

Early HBeAg-positive cohort							Late HBeAg-positive cohort						
Cutoff *	Sen (%)	Spe (%)	+LR	−LR	+PV (%)	−PV (%)	Cutoff *	Sen (%)	Spe (%)	+LR	−LR	+PV (%)	−PV (%)
HBsAg							HBsAg						
≤4.357 ^†^	72.1	60.2	1.81	0.46	91.8	25.9	≤4.571^†^	85.4	46.4	1.59	0.32	91.5	32.0
≤4.166 ^‡^	63.9	63.9	1.77	0.57	91.6	22.3	≤3.949^‡^	57.1	57.1	1.33	0.75	90.0	16.5
≤4.602 ^§^	82.6	45.8	1.52	0.38	90.4	29.9	≤4.602^§^	87.0	44.1	1.55	0.30	91.3	33.3
HBeAg							HBeAg						
≤3.062 ^†^	75.4	55.4	1.69	0.44	91.3	26.7	≤3.062^†^	74.1	53.6	1.60	0.48	91.5	23.4
≤2.885 ^‡^	60.7	60.2	1.53	0.65	90.4	19.9	≤2.744^‡^	56.1	56.0	1.27	0.78	89.6	15.9
≤3.114 ^§^	83.8	43.4	1.48	0.37	90.1	30.3	≤3.114^§^	80.3	39.3	1.32	0.50	89.9	22.8
Early HBeAg-positive sub-cohort with PHVR							Late HBeAg-positive sub-cohort with PHVR						
Cutoff *	Sen (%)	Spe (%)	+LR	−LR	+PV (%)	−PV (%)	Cutoff *	Sen (%)	Spe (%)	+LR	−LR	+PV (%)	−PV (%)
HBsAg							HBsAg						
≤4.254^†^	57.7	84.8	3.78	0.50	95.9	24.5	≤4.571^†^	77.9	76.5	3.31	0.29	96.1	32.0
≤4.496^‡^	68.4	67.8	2.12	0.47	92.9	25.8	≤4.549^‡^	76.6	76.5	3.26	0.31	96.0	30.7
≤4.602^§^	76.1	61.0	1.95	0.39	92.3	29.3	≤4.602^§^	80.3	72.6	2.93	0.27	95.6	33.3
HBeAg							HBeAg						
≤3.062^†^	65.9	76.3	2.78	0.45	94.5	26.6	≤3.062^†^	60.9	88.2	5.18	0.44	97.4	23.4
≤3.082^‡^	68.1	67.8	2.12	0.47	92.9	25.6	≤3.110^‡^	69.7	68.6	2.22	0.44	94.2	23.5
≤3.114^§^	77.2	59.3	1.90	0.38	92.1	29.7	≤3.114^§^	70.2	64.7	1.99	0.46	93.6	22.8
Early HBeAg-positive sub-cohort with PLVR							Late HBeAg-positive sub-cohort with PLVR						
Cutoff *	Sen (%)	Spe (%)	+LR	−LR	+PV (%)	−PV (%)	Cutoff *	Sen (%)	Spe (%)	+LR	−LR	+PV (%)	−PV (%)
HBV DNA							HBV DNA						
>3.775^†^	83.1	61.9	2.18	0.27	87.1	54.2	>3.963^†^	76.7	50.8	1.56	0.46	80.6	44.9
>4.223^‡^	69.2	69.1	2.24	0.45	87.4	42.0	>4.729^‡^	62.6	62.3	1.66	0.60	81.6	38.4
>3.699^§^	85.4	59.5	2.11	0.25	86.7	56.8	>3.903^§^	76.7	47.5	1.46	0.49	79.6	43.3
Early HBeAg-negative cohort							Late HBeAg-negative cohort						
Cutoff *	Sen (%)	Spe (%)	+LR	−LR	+PV (%)	−PV (%)	Cutoff *	Sen (%)	Spe (%)	+LR	−LR	+PV (%)	−PV (%)
HBV DNA							HBV DNA						
>3.758^†^	66.9	73.0	2.48	0.45	72.2	67.8	>3.886^†^	63.2	81.3	3.37	0.45	74.4	72.0
>3.575^‡^	69.4	69.2	2.25	0.44	70.2	68.3	>3.550^‡^	70.3	70.5	2.38	0.42	67.2	73.4
>3.301^§^	73.4	60.8	1.87	0.44	66.2	68.6	>3.301^§^	73.6	64.1	2.05	0.41	63.8	73.9
Early HBeAg-negative sub-cohort with PHSE							Late HBeAg-negative sub-cohort with PHSE						
Cutoff *	Sen (%)	Spe (%)	+LR	−LR	+PV (%)	−PV (%)	Cutoff *	Sen (%)	Spe (%)	+LR	−LR	+PV (%)	−PV (%)
HBV DNA							HBV DNA						
>4.678^†^	63.8	82.6	3.66	0.44	79.3	68.6	>3.846^†^	75.9	81.1	4.01	0.30	77.5	79.6
>4.196^‡^	70.3	70.5	2.38	0.42	71.3	69.4	>3.746^‡^	78.0	78.1	3.55	0.28	75.3	80.5
>4.000^§^	74.6	66.7	2.24	0.38	70.1	71.5	>3.699^§^	79.4	76.2	3.34	0.27	74.2	81.2
Early HBeAg-negative sub-cohort with PLSE							Late HBeAg-negative sub-cohort with PLSE						
Cutoff *	Sen (%)	Spe (%)	+LR	−LR	+PV (%)	−PV (%)	Cutoff *	Sen (%)	Spe (%)	+LR	−LR	+PV (%)	−PV (%)
HBV DNA							HBV DNA						
>3.636^†^	54.6	87.6	4.41	0.52	82.2	64.8	>3.897^†^	54.1	81.9	2.98	0.56	71.9	67.4
>2.817^‡^	64.6	64.8	1.83	0.55	65.7	63.6	>3.389^‡^	66.5	66.5	1.99	0.50	63.1	69.8
>2.699^§^	69.1	60.0	1.73	0.52	64.4	64.9	>3.301^§^	67.6	64.2	1.89	0.51	61.9	69.7

HBsAg, hepatitis B surface antigen; HBeAg, hepatitis B e antigen; HBV DNA, hepatitis B virus DNA; SHA, significant hepatitis activity; Sen, sensitivity; Spe, specificity; +LR, positive likelihood ratio; −LR, negative likelihood ratio; +PV, positive predictive value; −PV, negative predictive value; PHVR, possibly high HBV replication; PLVR, possibly low HBV replication; PHSE, possibly high HBsAg expression; PLSE, possibly low HBsAg expression.

* Units of measurement: HBsAg, log_10_ IU/ml; HBeAg, log_10_ SCO; HBV DNA, log_10_ IU/ml.

^†^ Optimal cutoff.

^‡^ Tradeoff cutoff.

^§^ Practical cutoff.

### Precision of hepatitis B surface antigen, hepatitis B e antigen, and hepatitis B virus DNA in predicting liver pathological conditions

With criteria of the practical cutoffs, the proportion of HBsAg, HBeAg, and HBV DNA alone and in tandem with ALT <40 IU/L in predicting liver pathological conditions in the HBeAg-positive and HBeAg-negative cohorts is summarized in **Table 5**.

**Table 5:** Proportion of HBsAg, HBeAg, and HBV DNA alone and in tandem with ALT in predicting pathological conditions.

Early HBeAg-positive cohort								
Variable *	N	≤G1 and ≤S1, % (n)	≤G1 and ≤S2, % (n)	≥G2, % (n)	≥G3, % (n)	≥S2, % (n)	≥S3, % (n)	≥S4, % (n)
ALT < 40	164	50.6 (83)	70.1 (115)	26.2 (43)	11.0 (18)	47.6 (78)	21.3 (35)	11.6 (19)
HBsAg > 4.602	129	51.2 (66)	70.5 (91)	29.5 (38)	10.1 (13)	42.6 (55)	9.3 (12)	5.4 (7)
HBsAg > 4.602 plus ALT < 40	53	71.7 (38)	94.3 (50)	5.7 (3)	1.9 (1)	28.3 (15)	1.9 (1)	0.0 (0)
HBeAg > 3.114	119	56.3 (67)	68.1 (81)	30.3 (36)	8.4 (10)	35.3 (42)	9.2 (11)	3.4 (4)
HBeAg > 3.114 plus ALT < 40	45	80.0 (36)	91.1 (41)	8.9 (4)	0.0 (0)	17.8 (8)	0.0 (0)	0.0 (0)
Late HBeAg-positive cohort								
Variable *	N	≤G1 and ≤S1, % (n)	≤G1 and ≤S2, % (n)	≥G2, % (n)	≥G3, % (n)	≥S2, % (n)	≥S3, % (n)	≥S4, % (n)
ALT < 40	174	48.3 (84)	69.5 (121)	27.0 (47)	6.9 (12)	49.4 (86)	21.8 (38)	13.8 (24)
HBsAg > 4.602	111	63.1 (70)	79.3 (88)	19.8 (22)	3.6 (4)	35.1 (39)	7.2 (8)	6.3 (7)
HBsAg > 4.602 plus ALT < 40	48	77.1 (37)	91.7 (44)	6.2 (3)	2.1 (1)	22.9 (11)	4.2 (2)	4.2 (2)
HBeAg ≤ 3.114	145	57.9 (84)	73.8 (107)	24.8 (36)	2.1 (3)	35.9 (52)	6.9 (10)	4.1 (6)
HBeAg ≤ 3.114 plus ALT < 40	44	75.0 (33)	93.2 (41)	4.5 (2)	0.0 (0)	22.7 (10)	2.3 (1)	2.3 (1)
Early HBeAg-negative cohort								
Variable	N	≤G1 and ≤S1, % (n)	≤G1 and ≤S2, % (n)	≥G2, % (n)	≥G3, % (n)	≥S2, % (n)	≥S3, % (n)	≥S4, % (n)
ALT < 40	284	58.5 (166)	77.1 (219)	16.5 (47)	7.4 (21)	40.8 (116)	18.3 (52)	10.2 (29)
HBV DNA ≤ 3.301	209	59.8 (125)	73.7 (154)	19.6 (41)	10.0 (21)	38.8 (81)	21.1 (44)	13.4 (28)
HBV DNA ≤ 3.301 plus ALT < 40	165	65.5 (108)	79.4 (131)	13.3 (22)	7.9 (13)	33.9 (56)	17.6 (29)	10.3 (17)
Late HBeAg-negative cohort								
Variable *	N	≤G1 and ≤S1, % (n)	≤G1 and ≤S2, % (n)	≥G2, % (n)	≥G3, % (n)	≥S2, % (n)	≥S3, % (n)	≥S4, % (n)
ALT < 40	441	63.9 (282)	82.3 (363)	14.1 (62)	3.2 (14)	35.4 (156)	14.3 (63)	7.7 (34)
HBV DNA ≤ 3.301	329	65.7 (216)	81.5 (268)	15.2 (50)	4.3 (14)	32.8 (108)	14.3 (47)	8.5 (28)
HBV DNA ≤ 3.301 plus ALT < 40	270	68.5 (185)	86.3 (233)	10.0 (27)	2.2 (6)	31.1 (84)	11.5 (31)	6.7 (18)

ALT, alanine transferase; HBsAg, hepatitis B surface antigen; HBeAg, hepatitis B e antigen; HBV DNA, hepatitis B virus DNA; Gx (x = 0, 1, 2, 3 or 4), level of pathological grade; Sy (y = 0, 1, 2, 3 or 4), level of pathological stage.

* Units of measurement: ALT, IU/L; HBsAg, log_10_ IU/ml; HBeAg, log_10_ SCO; HBV DNA, log_10_ IU/ml.

## Discussion

Each phase of the natural history does not yet have a rational denomination (30). The first phase of the natural history has always been considered an immune tolerance, but this designation has been challenged in recent years (21–33). Patients in the ‘immune tolerance’ phase are rarely free of liver necro-inflammation (26, 31). Therefore, this study refers to the four natural history phases from a clinical perspective as HBeAg-positive NSHA and SHA and HBeAg-negative NSHA and SHA.

Accurate delimitation of the natural history phases remains a clinical challenge (3–6, 26, 34). As a sensitive indicator, ALT does not necessarily reflect liver injury that has occurred; conversely, as specific indicators, Grade and Stage do not necessarily reflect liver injury that is occurring. In the absence of evidence of liver pathology, relying only on high levels of HBV DNA and normal levels of ALT, even with a fixed period of regular follow-up, cannot rule out the possibility of NSHA in HBeAg-positive patients. In fact, the oscillations between adjacent phases are often missed during regular follow-up, especially between HBeAg-positive NSHA and SHA. In addition, the division point between high and non-high levels of HBV DNA in delimiting HBeAg-positive NSHA and SHA remains controversial (3–6); the upper limit of normal reference for ALT is also continuously debated and updated (35), although ALT <40 IU/L is generally considered normal. Therefore, the accurate delimitation of the natural history phases requires the development and evaluation of new laboratory indicators.

The LOESS regression analyses in the HBeAg-positive and HBeAg-negative cohorts showed evolving correlations between ALT and HBsAg, HBeAg, and HBV DNA. In the HBeAg-positive cohorts, ALT displayed clearly negative correlations with high levels of HBsAg, HBeAg, and HBV DNA but illustrated possibly divergent correlations with moderate-to-low levels of HBsAg, HBeAg, and HBV DNA. In the HBeAg-negative cohorts, ALT illustrated clearly divergent correlations with HBsAg and HBV DNA. Spearman’s correlation analyses in the HBeAg-positive and HBeAg-negative sub-cohorts showed similar findings to the LOESS regressions. In addition, in the HBeAg-positive sub-cohorts with PHVR, HBsAg, HBeAg, and HBV DNA produced significantly negative correlations with Grade and Stage; however, in sub-cohorts with PLVR, ALT produced divergent correlations with Grade and Stage. In the HBeAg-negative sub-cohorts with PHSE, HBsAg and HBV DNA indicated significantly negative and positive correlations with Grade and Stage, respectively; however, in sub-cohorts with PLSE, they indicated significant positive correlations with Stage.

The findings from the LOESS regression and Spearman’s correlation analyses suggested that HBsAg, HBeAg, and HBV DNA have divergent signatures in the evolution of the natural history, which indirectly supported some inferences proposed by some experimental studies (19–21). HBsAg may dampen the host’s immune activation in a concentration–response relationship; high levels of HBeAg may synergize with HBsAg to dampen the immune activation, and medium-to-low levels of HBeAg may sharpen the immune activation; HBV replication not only dampens the immune activation by increasing HBsAg and HBeAg production but also sharpens the immune activation by increasing HBcrAg production including HBeAg. These findings also suggested that the evolution of the natural history results primarily from the age-related progressive decline in HBV replication levels and the progressive decline in HBsAg and HBeAg production (31). In this study, the patients in the HBeAg-positive sub-cohorts with PHVR were significantly younger and had significantly higher HBsAg and HBeAg levels than those with PLVR, and in the HBeAg-negative sub-cohorts with PHSE, the patients were also significantly younger and had significantly higher HBV DNA levels than those with PLSE.

The ROC curve analyses of this study showed that, in predicting SHA in the HBeAg-positive sub-cohorts with PHVR and PLVR, HBsAg was moderately accurate and ‘less accurate or uninformative’ in the same test directions, respectively; HBeAg was moderately accurate and ‘less accurate or uninformative’ in the opposite test directions, respectively; HBV DNA was less accurate and ‘moderately or less accurate’ in the opposite test directions, respectively. In predicting SHA in the HBeAg-negative sub-cohorts with PHSE and PLSE, HBsAg was ‘less accurate or uninformative’ and uninformative in the opposite test directions, respectively; HBV DNA was moderately accurate and moderately accurate in the same test directions, respectively. In predicting SHA in the early and late HBeAg-positive cohorts, their HBsAg was less accurate in the same test directions; in the early and late HBeAg-negative cohorts, their HBV DNA was moderately accurate in the same test directions.

The findings from the ROC curve analyses suggested that the ability of HBsAg to dampen immune activation remains dominant and gradually weakens from HBeAg-positive PHVR and PLVR to HBeAg-negative PHSE and may be lost in HBeAg-negative PLSE; the ability of HBeAg to dampen immune activation remains dominant only in patients with HBeAg-positive PHVR and reverses in PLVR. These findings also suggested that HBsAg instead of HBV DNA in predicting HBeAg-positive SHA and HBV DNA instead of HBsAg in predicting HBeAg-negative SHA are valuable. However, the power of HBsAg in predicting HBeAg-positive and HBV DNA in predicting HBeAg-negative SHA is not strong enough, which needs to be made up in tandem with other non-invasive laboratory parameters. Therefore, this study proposed practical cutoffs for HBsAg, HBeAg, and HBV DNA that gave priority to sensitivity.

With criteria of the practical cutoffs, this study further evaluated the precision of HBsAg, HBeAg, and HBV DNA alone and in tandem with ALT in predicting liver pathological conditions. The results showed that in the HBeAg-positive cohorts, HBsAg or HBeAg in tandem with ALT was excellent in both defining HBeAg-positive ‘minimal necro-inflammation and non-extensive fibrosis (Grade ≤G1 and Stage ≤S2) and excluding HBeAg-positive extensive fibrosis (Stage ≥S3) and cirrhosis (Stage ≥S4); however, in the HBeAg-negative cohorts, HBV DNA in tandem with ALT was good in both defining HBeAg-negative ‘minimal necro-inflammation and non-extensive fibrosis’ and excluding HBeAg-negative extensive fibrosis and cirrhosis. These findings suggested that HBsAg or HBeAg in tandem with ALT is very useful and has a very low clinical risk in defining HBeAg-positive NSHA and SHA; however, HBV DNA in tandem with ALT is merely usable and has a very high clinical risk in defining HBeAg-negative NSHA and SHA (26, 34).

This study has some limitations. First, it used a cross-sectional design; however, it is impractical and unethical to regularly follow up patients with liver pathology data for a fixed short period of time. Second, it did not address other novel HBV markers; however, it is a real-world study with large-scale samples and evaluated in detail the usability of HBsAg and HBV DNA, which are currently accessible HBV markers. Third, it involved only adult patients; its conclusions may not necessarily apply to pediatric patients.

In summary, this study discovered for the first time that the ROC curves of HBV DNA or HBV DNA and HBeAg in predicting SHA in the entire HBeAg-positive cohorts and those of HBsAg in predicting SHA in the entire HBeAg-negative cohorts behave as a ‘biphasic shape’. On this basis, this study designated the HBeAg-positive sub-cohorts with PHVR and PLVR, and HBeAg-negative sub-cohorts with PHSE and PLSE; it proved for the first time the usability of HBsAg instead of HBV DNA in delimiting HBeAg-positive NSHA and SHA and of HBV DNA instead of HBsAg in delimiting HBeAg-negative NSHA and SHA.

## Data availability statement

The original contributions presented in the study are included in the article/supplementary material. Further inquiries can be directed to the corresponding author.

## Ethics statement

The studies involving human participants were reviewed and approved by ethics committee of Shanghai Public Health Clinical Center. Written informed consent to participate in this study was provided by the participants’ legal guardian/next of kin.

## Author contributions

ZZ was a involved in study concept and design, securing research funding, acquisition of data, analysis and interpretation of data, and drafting of the manuscript. WL and DH were involved in the collation of clinical, laboratory, and pathological data. WL, DH, XZ, RD, XL, YW, and WJL were involved in the acquisition of clinical and laboratory data. DZ and YF were involved in the acquisition of pathological data. WL and DH were involved in the critical revision of the manuscript. All authors have read and agreed to the published version of the manuscript.

## Funding

This work was partly funded by the ‘12th Five-year’ and the ‘13th Five-year’ National Science and Technology Major Project of China (2013ZX10002005, 2017ZX10203202).

## Conflict of interest

The authors declare that the research was conducted in the absence of any commercial or financial relationships that could be construed as a potential conflict of interest.

The reviewer JG declared a shared parent affiliation with authors to the handling editor at the time of review.

## Publisher’s note

All claims expressed in this article are solely those of the authors and do not necessarily represent those of their affiliated organizations, or those of the publisher, the editors and the reviewers. Any product that may be evaluated in this article, or claim that may be made by its manufacturer, is not guaranteed or endorsed by the publisher.
